# Oral Anticoagulant Use Among Older Adults in Long‐Term Care Facilities: Trends Over Time and Impact of High‐Risk Comorbidities

**DOI:** 10.1002/pds.70356

**Published:** 2026-03-30

**Authors:** S. L. Harrison, M. C. Inacio, T. Air, C. Lang, J. K. Sluggett, C. Whitehead, G. E. Caughey

**Affiliations:** ^1^ Registry of Senior Australians Research Centre, Caring Futures Institute, College of Nursing and Health Sciences Flinders University Bedford Park South Australia Australia; ^2^ Registry of Senior Australians Research Centre South Australian Health and Medical Research Institute Adelaide South Australia Australia; ^3^ UniSA Allied Health and Human Performance University of South Australia Adelaide South Australia Australia; ^4^ College of Medicine and Public Health Flinders University Adelaide Australia; ^5^ Southern Adelaide Local Health Network SA Health Adelaide South Australia Australia

**Keywords:** anticoagulants, long‐term care facilities, population‐based study

## Abstract

**Purpose:**

To examine oral anticoagulant (OAC) use in older adults residing in long‐term care facilities (LTCFs) since direct OACs (DOACs) were introduced, including trends over time and in people with high‐risk comorbidities (prior gastrointestinal bleed and dementia).

**Methods:**

A repeated cross‐sectional study using the Registry of Senior Australians National Historical Cohort was conducted and included individuals aged ≥ 65 years residing in LTCFs in Australia between 2013 and 2019. Yearly age‐ and sex‐adjusted prevalence of OACs and 95% confidence intervals (CIs) were estimated using generalised estimating equation (GEE) Poisson models and adjusted prevalence ratios (aPRs) were calculated to estimate trends.

**Results:**

In 500 883 individuals, the prevalence of OAC use increased from 12.2% (95% CI 12.1–12.4) in 2013 to 16.5% (95% CI 16.4–16.7) in 2019 [aPR 1.05 (95% CI 1.05–1.05)]. This was driven by an increase in the use of DOACs, from 2.24% (95% CI 2.19–2.29) to 12.5% (95% CI 12.3–12.6) [aPR 1.25 (95% CI 1.25–1.26)]. Increases in OAC use were also observed in people with prior gastrointestinal bleed, from 12.9% (95% CI 11.8–13.9) to 17.1% (95% CI 16.2–17.9) [aPR 1.05 (95% CI 1.03–1.06)]. The use of OACs increased over the study period for people living with dementia from 9.93% to 12.8% [aPR 1.04 (95% CI 1.04–1.05)], and for people without dementia from 15.7% to 21.7% [aPR 1.06 (95% CI 1.06–1.06)].

**Conclusions:**

This population‐based study showed increasing use of OACs among residents of LTCFs, including in higher‐risk comorbidity groups, indicating growing confidence in the use of DOACs in these groups.

## Key Points

- Little is known regarding oral anticoagulant (OAC) use by older people living in long‐term care facilities that have high‐risk comorbidities that are linked with an increased bleeding risk
- In a national study of over 500 000 people in long‐term care facilities 2013–2019, OAC use increased including in potentially high‐risk groups including those with prior gastrointestinal bleed and dementia.
- The increase in OAC use was driven by an increase in direct OACs.
- There may be room for improvement in the prescription of OACs in long‐term care facilities as uncertainty may remain among prescribing physicians when determining whether to prescribe or deprescribe OACs in older people with complex health care needs.

## Plain Language Summary

Oral anticoagulants are used to reduce the risk of blood clots and strokes in people at risk of these conditions. Since 2013, newer anticoagulants became available, offering benefits over older medications like warfarin, including improved effectiveness, lower bleeding risk and less intensive monitoring. Older adults living in long‐term care facilities (nursing homes) often have multiple health conditions and more than half are living with dementia. Certain conditions such as dementia or a history of bleeding may complicate decisions about whether to prescribe oral anticoagulants. This study aimed to examine the use of oral anticoagulants in long‐term care facilities since the introduction of the newer oral anticoagulants. We analysed national data from Australia, including over 500 000 people aged over 65 years in long‐term care facilities from 2013 to 2019. Our findings showed an increase in the use of oral anticoagulants from 12% to 17% of residents. This rise was mainly due to an increased use of newer anticoagulants, with a decrease in warfarin use. The shift toward newer medications was seen in people with high‐risk conditions such as a history of gastrointestinal bleeding and dementia. However, people living with dementia were still less likely to be treated with oral anticoagulants overall.

## 1 Introduction

Oral anticoagulants (OACs) are strongly recommended for primary or secondary stroke prevention in patients with non‐valvular atrial fibrillation (AF) and a CHA_2_DS_2_‐VA stroke risk score of two or more, unless contraindicated [1, 2]. OACs are also indicated in the treatment of most cases of venous thromboembolism (VTE) to prevent recurrence, including in the treatment of pulmonary embolism (PE) [3]. Since their introduction in 2013, direct OACs (DOACs) have become the preferred treatment option globally for most cases of non‐valvular AF, VTE and PE in preference to warfarin, due to their comparable safety and efficacy, without the need for frequent blood monitoring as required for OACs such as warfarin [1, 2, 3, 4, 5, 6].

OACs can increase the risk of bleeding, but in the majority of patients with indications for use, the beneficial impact of stroke prevention outweighs the increased risk of bleeding [4]. Previous observational studies of older people have suggested that factors associated with an increased risk of bleeding, such as dementia or prior gastrointestinal (GI) bleed may also be associated with reduced likelihood of receiving OACs [7, 8, 9, 10, 11]. Yet, real‐world evidence has suggested the beneficial impact of DOACs in reducing the risk of stroke continues to outweigh the risk of bleeding in the older population, including those with polypharmacy, multimorbidity, frailty and dementia [8, 12, 13].

Older people living in long‐term care facilities (LTCFs; also known as nursing homes, care homes, residential aged care) are often aged over 80 years, have multimorbidity, polypharmacy, frailty and cognitive impairment and are commonly excluded from randomised controlled trials [14, 15]. Prevalence estimates of OAC use among older people living in LTCFs vary by population characteristics; previous studies have often focused exclusively on residents with AF, with estimates of OAC use varying widely between 17% and 63% [16]. In a large US study of over 500 000 nursing home residents, 12% received OACs and among those treated, 44% were prescribed DOACs [17]. However, this estimate was from 2016 and may not reflect more recent OAC prescribing patterns in LTCFs. There has been limited evidence of the use of OACs in people living in LTCFs, but observational evidence in the United Kingdom and the United States has suggested treatment could likely be improved for certain individuals [7, 10].

In this national study of older individuals living in LTCFs, the primary objectives were to examine trends in use of OACs since the introduction of DOACs and by high‐risk comorbidities which may influence OAC prescribing decisions (i.e., dementia and prior GI bleeding). The secondary objectives were to determine the use of OACs in long term care residents by indication including AF, VTE, PE and ischaemic stroke, and examine concurrent trends in the use of other antithrombotic medicines (i.e., antiplatelets, heparin).

## 2 Methods

### 2.1 Study Design and Data Source

A repeated cross‐sectional study between 2013 and 2019 was conducted using the Registry of Senior Australians (ROSA) National Historical Cohort [14]. The ROSA National Historical Cohort contains integrated national health, aged care and social welfare data for ~3.5 million Australians who have accessed aged care services between 2002 and 2020. In Australia, universal health care and government subsidies for long‐term care services exist, with most individuals accessing health and long‐term care services through the programs captured within the employed datasets [18, 19]. For this analysis, datasets used included national long‐term care eligibility assessments and care assessments at entry to LTCFs; national pharmaceutical dispensing records (i.e., Pharmaceutical Benefits Scheme [PBS]); inpatient hospitalisation and emergency department (ED) presentations for four out of seven states and major territories (87% of the national cohort); and death records (National Death Index).

### 2.2 Study Cohort

The cohort included individuals residing in LTCFs between 1 January 2013 and 31 December 2019, aged between ≥ 65 years and ≤ 105 years at cohort entry and resided in South Australia, Queensland, New South Wales, or Victoria. LTCFs in Australia provide 24‐h care and accommodation for older people who can no longer live at home; most residents have complex health needs, with approximately 60% experiencing five or more comorbidities, and over half will have dementia [20]. Aboriginal and Torres Strait Islander people were not included due to the requirement for leadership from Aboriginal and Torres Strait Islander communities and specific governance and ethical approvals, which were beyond the scope of the current project. Cohort entry was defined as 1 January 2013 for existing residents, or the date of entry to a LTCF for those who entered after that date.

### 2.3 Exposure

The primary exposure was the annual prevalence of use of OACs including warfarin (Anatomical Therapeutic Chemical [ATC] code B01AA03) or DOACs (rivaroxaban [B01AF02], dabigatran [B01AE07] and apixaban [B01AF01]). OAC use was defined as having at least one dispensing record during the calendar year. For analyses of OAC use by calendar year, individuals were included only if they were residents of a LTCF for at least 1 day during that year. Specifically, individuals who newly entered a LTCF during the year were added to the denominator, while those who died before the start of the year were excluded. The use of heparin (B01AB) and antiplatelets (B01AC) was also examined. In addition to examining all antiplatelets, two sensitivity analyses were conducted: one examining antiplatelets excluding single‐ingredient aspirin products, and another examining aspirin alone, as aspirin was delisted from Australia's national medicines subsidy scheme on 1 January 2016.

### 2.4 Covariates

Cohort characteristics were determined from aged care assessment data including age, sex, country of birth (Australia or other), state of residence and remoteness classification based on the Australian Statistical Geography Standard [21] (Table 1). Number of chronic conditions were calculated using the pharmaceutical based Rx‐Risk‐V Comorbidity Index [22] (excluding the OAC group) and categorised into 0–4, 5–6 or ≥ 7 comorbid conditions in the 6 months prior to cohort entry. Hospitalisation records based on admissions from 12 months before cohort entry to the end of follow‐up were used to identify any previous history of AF (International Classification of Diseases and Related Health Problems, Tenth Revision, Australian Modification (ICD‐10‐AM) code I48*), VTE (ICD‐10‐AM codes I80*, I81*, I82* and I26*), PE (ICD‐10‐AM code I26*), ischaemic stroke (ICD‐10‐AM codes I63*) and GI bleed (Table S1). Conditions were coded as absent until first recorded and present thereafter. Dementia status was determined using PBS data for the 6 months prior to cohort entry in addition to hospitalisation records, aged care eligibility assessments and assessments at entry to a LTCF [23].

**TABLE 1:** Cohort characteristics by oral anticoagulant status.

Characteristic	Total cohort (*n* = 500 883)	Received OAC (*n* = 85 990)	No OAC (*n* = 414 893)
Female	315 658 (63.0)	52 459 (61.0)	263 199 (63.4)
Age at cohort entry (years), median (IQR)	86 (80–90)	85 (81–89)	86 (80–90)
≤ 74	52 344 (10.5)	7544 (8.8)	44 800 (10.8)
75–84	162 392 (32.4)	30 240 (35.2)	132 152 (31.9)
85–94	250 075 (49.9)	44 797 (52.1)	205 278 (49.5)
≥ 95	36 072 (7.2)	3409 (4.0)	32 663 (7.9)
Born in Australia	348 669 (69.6)	61 152 (71.1)	287 517 (69.3)
Missing	1230 (0.2)	227 (0.3)	1003 (0.2)
State			
New South Wales	193 109 (38.6)	34 262 (39.8)	158 847 (38.3)
Queensland	104 966 (21.0)	17 383 (20.2)	87 583 (21.1)
South Australia	52 047 (10.4)	9543 (11.1)	42 504 (10.2)
Victoria	150 761 (30.1)	24 802 (28.8)	125 959 (30.4)
Remoteness			
Major cities	349 024 (69.7)	60 140 (69.9)	288 884 (69.6)
Inner regional	113 609 (22.7)	19 493 (22.7)	94 116 (22.7)
Outer regional	35 192 (7.0)	5845 (6.8)	29 347 (7.1)
Remote/very remote	1678 (0.4)	228 (0.3)	1450 (0.3)
Missing	1380 (0.3)	284 (0.3)	1096 (0.3)
Comorbid conditions			
Dementia	292 357 (58.4)	40 637 (47.3)	251 720 (60.7)
Atrial fibrillation^a^	60 018 (12.0)	27 323 (31.8)	32 695 (7.9)
Ischaemic stroke^a^	17 818 (3.6)	6387 (7.4)	11 431 (2.8)
Pulmonary embolism^a^	5038 (1.0)	3438 (4.0)	1600 (0.4)
Venous thromboembolism^a^	11 771 (2.4)	6749 (7.8)	5022 (1.2)
Gastrointestinal bleed^a^	14 628 (2.9)	3208 (3.7)	11 420 (2.8)
Comorbidity score^b^, median (IQR)	5 (3–7)	6 (4–7)	5 (3–7)
0–4 conditions	208 371 (41.6)	26 794 (31.2)	181 577 (43.8)
5–6 conditions	145 640 (29.1)	27 713 (32.2)	117 927 (28.4)
≥ 7 conditions	146 872 (29.3)	31 483 (36.6)	115 389 (27.8)

*Note:* Characteristics of people living in long‐term care facilities, 1 January 2013 to 31 December 2019. All values % (*n*), unless otherwise specified. Cohort entry was defined as 1 January 2013 for existing residents, or the date of entry to a long‐term care facility for those who entered after that date.

^a^ Based on hospitalisation data.

^b^ Rx‐Risk‐V pharmaceutical based comorbidity index excluding oral anticoagulants.

### 2.5 Statistical Analysis

Cohort characteristics were described overall and by those who did and did not receive an OAC during the study period using medians and interquartile ranges for continuous variables and frequencies and percentages for categorical variables. Yearly age and sex adjusted OAC and antiplatelet prevalence and 95% confidence intervals (CIs) were estimated using generalised estimating equation (GEE) Poisson models. All models were estimated with robust standard errors to account for the non‐independence of observations and exchangeable correlation structure. Age and sex adjusted prevalence ratios (aPRs) to estimate linear trends were calculated using GEE Poisson models. All analyses were completed using SAS version 9.4 (SAS Institute Inc) and Stata version 18.0 (StataCorp 2023).

## 3 Results

### 3.1 Participant Characteristics

A total of 500 883 people residing in LTCFs were included. Of these individuals, 63.0% (*n* = 315 658) were female, the median (interquartile range: IQR) age was 86 (80–90) years, 58.4% (*n* = 292 357) were living with dementia and 2.9% (*n* = 14 628) had a prior GI bleed.

Conditions with indications for OAC use included: history of AF (12.0%, *n* = 60 018), VTE (2.4%, *n* = 11 771), PE (1.0%, *n* = 5038) and ischaemic stroke (3.6%, *n* = 17 818). The median (IQR) Rx‐Risk medication‐based comorbidity index score was 5 (3–7). Overall, 17.2% (*n* = 85 990) were dispensed an OAC during the study period.

### 3.2 Changes in Use of OACs Over Time

**FIGURE 1:**
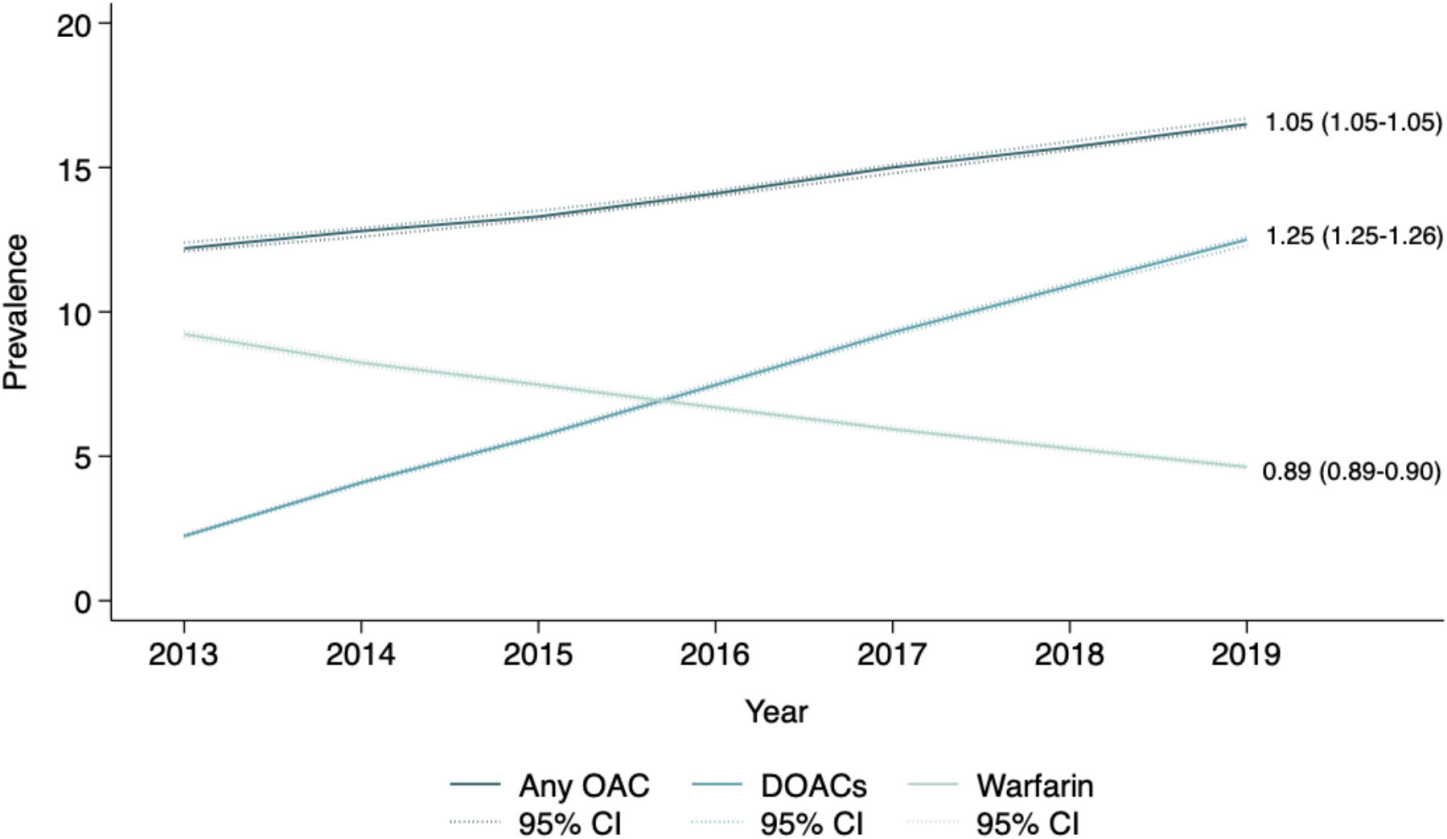
Yearly prevalence of oral anticoagulants use and prevalence ratio over the study period. Values displayed at the end of each line are adjusted prevalence ratios and 95% CIs. CI, confidence interval; DOAC, direct oral anticoagulant; OAC, oral anticoagulant.

Between 2013 and 2019, the proportion of people who were dispensed an OAC increased from 12.2% (95% CI 12.1–12.4) in 2013 to 16.5% (95% CI 16.4–16.7) in 2019. There was an increase in the use of OACs by approximately 5% per calendar year (aPR 1.05 95% CI 1.05–1.05) (Table 2, Figure 1). The prevalence of DOACs increased over time from 2.24% (95% CI 2.19–2.29) in 2013 to 12.5% (95% CI 12.3–12.6) in 2019 [aPR 1.25 (95% CI 1.25–1.26)]. In the study period, the prevalence of warfarin decreased from 9.23% (95% CI 9.11–9.34) in 2013 to 4.63% (95% CI 4.56–4.71) in 2019 [aPR 0.89 (95% CI 0.89–0.90)]. The prevalence of other antithrombotics, including antiplatelets and heparin, also decreased over time [aPRs 0.83 (95% CI 0.82–0.83) and 0.96 (95% CI 0.96–0.97), respectively]. After excluding aspirin, the prevalence of antiplatelet use also declined over time [aPR 0.96 (95% CI 0.96–0.97)].

**TABLE 2:** Prevalence (95% confidence interval) of anticoagulants and antiplatelets by year adjusted by age and sex.

Total cohort (*N* = 500 883)	2013	2014	2015	2016	2017	2018	2019	Adjusted prevalence ratio
	*N* = 190 863	*N* = 193 061	*N* = 195 463	*N* = 197 532	*N* = 200 575	*N* = 200 515	*N* = 203 284	
Any OAC	12.2 (12.1–12.4)	12.8 (12.6–12.9)	13.3 (13.2–13.5)	14.1 (14.0–14.2)	15.0 (14.8–15.1)	15.7 (15.6–15.9)	16.5 (16.4–16.7)	1.05 (1.05–1.05)
Warfarin	9.23 (9.11–9.34)	8.24 (8.14–8.34)	7.48 (7.39–7.57)	6.69 (6.60–6.77)	5.94 (5.86–6.02)	5.27 (5.19–5.36)	4.63 (4.56–4.71)	0.89 (0.89–0.90)
DOAC	2.24 (2.19–2.29)	4.09 (4.02–4.16)	5.69 (5.61–5.77)	7.47 (7.38–7.57)	9.29 (9.18–9.39)	10.9 (10.8–11.0)	12.5 (12.3–12.6)	1.25 (1.25–1.26)
Rivaroxaban	1.06 (1.03–1.10)	1.98 (1.93–2.03)	2.59 (2.53–2.65)	3.12 (3.06–3.19)	3.46 (3.40–3.53)	3.69 (3.62–3.76)	3.88 (3.80–3.95)	1.16 (1.16–1.17)
Dabigatran	0.50 (0.47–0.52)	0.77 (0.73–0.80)	0.78 (0.75–0.81)	0.86 (0.82–0.89)	0.99 (0.96–1.03)	1.05 (1.01–1.09)	1.04 (1.00–1.08)	1.10 (1.09–1.11)
Apixaban	0.51 (0.49–0.53)	1.22 (1.19–1.26)	2.27 (2.22–2.32)	3.57 (3.51–3.64)	5.03 (4.95–5.1)	6.49 (6.40–6.58)	7.92 (7.82–8.03)	1.40 (1.39–1.40)
Heparin	4.19 (4.11–4.28)	4.14 (4.05–4.22)	4.07 (3.99–4.16)	3.93 (3.85–4.02)	3.87 (3.79–3.95)	3.54 (3.46–3.62)	3.24 (3.17–3.32)	0.96 (0.96–0.97)
Antiplatelets	41.6 (41.3–41.8)	38.6 (38.4–38.8)	35.7 (35.6–35.9)	21.9 (21.8–22.1)	18.2 (18.0–18.3)	16.5 (16.4–16.7)	15.2 (15.1–15.4)	0.83 (0.82–0.83)
Antiplatelets (no aspirin)	11.8 (11.6–11.9)	11.1 (10.9–11.2)	10.4 (10.3–10.6)	10.9 (10.8–11.0)	10.3 (10.2–10.4)	9.68 (9.57–9.79)	9.08 (8.97–9.19)	0.96 (0.96–0.97)
Aspirin	29.7 (29.5–29.9)	27.6 (27.5–27.8)	25.4 (25.3–25.6)	11.2 (11.1–11.4)	8.05 (7.93–8.16)	7.01 (6.91–7.12)	6.26 (6.15–6.36)	0.74 (0.74–0.75)

Abbreviations: DOAC, direct oral anticoagulant; OAC, oral anticoagulant.

Of the individual type of DOACs, the largest increase in use was apixaban from 0.51% (95% CI 0.49–0.53) in 2013 to 7.92% (95% CI 7.82–8.03) in 2019 (aPR 1.40 95% CI 1.39–1.40), followed by rivaroxaban from 1.06% (95% CI 1.03–1.10) in 2013 to 3.88% (95% CI 3.80–3.95) in 2019 [aPR 1.16 (95% CI 1.16–1.17)] and dabigatran from 0.50% (95% CI 0.47–0.52) in 2013 to 1.04% (95% CI 1.00–1.08) in 2019 [aPR 1.10 (95% CI 1.09–1.11)].

### 3.3 Use of OACs in People Living With Dementia

In 2013, use of OACs among people with dementia was lower than for people without dementia [9.93% (95% CI 9.79–10.1) and 15.7% (95% CI 15.4–15.9), respectively]. The use of OACs increased over time for people with and without dementia [aPRs 1.04 (95% CI 1.04–1.05) and 1.06 (95% CI 1.06–1.06), respectively]. However, the prevalence of OACs remained lower for people with dementia than for people without dementia [2019 estimates: 12.8% (95% CI 12.6–12.9) for people with dementia and 21.7% (95% CI 21.4–21.9) for people without dementia]. The use of DOACs increased and warfarin decreased for both individuals with and without dementia (Figure S1, Table S2).

### 3.4 Use of OACs in People With Prior GI Bleeding

The use of OACs increased in people with and without prior GI bleeding (Table S3). The prevalence of OACs increased from 12.9% (95% CI 11.8–13.9) in 2013 to 17.1% (95% CI 16.2–17.9) in 2019 in people with a prior GI bleed [aPR 1.05 95% CI (1.03–1.06)] and from 12.1% (95% CI 12.0–12.2) to 16.5% (95% CI 16.4–16.7) in people without a prior GI bleed [aPR 1.05 (95% CI 1.05–1.06)].

### 3.5 Use of OACs by People With AF, Ischaemic Stroke, VTE and PE

The use of OACs increased over time in people with AF from 35.2% (95% CI 34.5–35.9) in 2013 to 42.9% (42.3–43.4) in 2019 [aPR 1.03 (95% CI 1.03–1.04)] (Table S4). No statistically significant change in the prevalence of OACs for people with ischaemic stroke was observed [aPR 1.00 (95% CI 1.00–1.01)], but there was an increase in the use of DOACs [aPR 1.12 (95% CI 1.11–1.13)] and a decrease in the use of warfarin [aPR 0.87 (95% CI 0.86–0.89)] (Table S5). Decreases in the use of OACs were observed in people with prior VTE from 55.8% (95% CI 53.7–57.9) in 2013 to 41.6% (95% CI 40.3–42.8) in 2019 [aPR 0.96 (95% CI 0.95–0.96)] (Table S6), and in people with prior PE from 68.7% (95% CI 65.3–72.0) in 2013 to 51.6% (95% CI 49.7–53.5) in 2019 [aPR 0.96 (95% CI 0.95–0.97)] (Table S7). However, the use of DOACs increased and the use of warfarin also decreased in people with prior VTE and in people with prior PE.

## 4 Discussion

In this study of over half a million older individuals who resided in LTCFs between 2013 and 2019, the use of OACs increased by 5% annually to 16.5%. The increase was largely driven by an increase in DOAC use (25% annually), which was accompanied by an 11% annual decrease in the use of warfarin and small decreases in antiplatelet use (4%, excluding aspirin) and heparin (4%). These findings suggest that DOACs have been increasingly prescribed to older, frail patients residing in LTCFs. Increases in OAC use were also observed in individuals with high‐risk comorbidities including prior GI bleed, potentially reflective of increased safety of DOACs in terms of GI bleeding risk [24]. Although increases in OAC use were observed in people living with dementia, their use remained lower compared to those without dementia, potentially highlighting the ongoing challenge that clinicians face when balancing the risks and benefits of OAC therapy for people with dementia.

The increases in OAC utilisation likely reflect greater clinician awareness of the benefits of OACs. However, in people living in LTCFs, polypharmacy remains a significant concern and the potential risks associated with multiple medications must be carefully managed alongside efforts to optimise OAC use. Despite increases in the proportion of people with AF receiving OACs, 57% of residents with AF did not receive an OAC in 2019. As the majority (89.5%) of the current study cohort were aged 75 years and over, the CHA_2_DS_2_‐VA score for most people with AF in the cohort would be at least 2 and therefore OACs would usually be recommended for stroke prevention [1]. The results are in line with similar findings in US nursing home residents with AF where an increase in OAC use from 42% in 2011 to 48% in 2016 was observed, but over half of individuals were still not receiving these therapies [25]. These are lower estimates than a similar study of care home residents in Wales which showed OAC use had increased to 73% in 2018, but that study included new residents only [7]. The results are also comparable to a large study predominately of older individuals (aged ≥ 80 years) in the US with AF where an increase in the use of OACs between 2011 and 2019 was observed (from 32% in 2011 to 44% in 2019), but the study was not exclusively in people living in LTCFs [8].

Clinicians may be cautious when prescribing OACs to residents of LTCFs due to several factors that complicate the risk–benefit assessment such as higher risk of falls and high prevalence of frailty, cognitive impairment, polypharmacy and limited life expectancy [26, 27]. For instance, every quarter, approximately 31% of residents in LTCFs in Australia experience a fall, and 14% require a hospitalisation due to a fall annually [28, 29]. Over half (58%) of the population in this study were living with dementia, and while utilisation of OACs increased for those with dementia over the study period, in 2019 the prevalence of OAC use in people with dementia was 12.8%. Current clinical guidelines suggest that there are very few absolute contraindications to OAC use, especially DOACs [2]. The presence of dementia is not a contraindication to OAC use [30, 31], yet people living with dementia are often at a higher risk of falls which may complicate treatment decisions [32]. Observational studies of the use of OACs in people living with dementia are important as these individuals were underrepresented in randomised controlled trials in people with non‐valvular AF and VTE comparing DOACs and warfarin [24, 33, 34, 35, 36, 37, 38]. DOACs offer a potentially safer alternative to warfarin in people living with dementia in terms of bleeding risk, less variation in anticoagulation levels and need for monitoring [30, 31]. In an observational study of > 1.1 million older adults with AF in the US comparing the use of different types of OACs, the absolute net clinical benefits associated with apixaban in comparison with other OACs was greater among persons living with dementia than those without dementia [31]. A patient‐centred approach should be taken when prescribing OACs to older people living with dementia or with a high bleeding risk score, to allow the person or their family members to make an informed decision [4].

No previous randomised controlled trials of OACs have been conducted exclusively with older people in LTCFs. Previous observational studies examining OAC use of older people with AF in LTCFs in the US have suggested the benefits of OACs to reduce the risk of stroke and mortality outweigh the risk of bleeding in this population, and the use of DOACs may be associated with a lower risk of adverse outcomes compared to warfarin [39, 40]. However, observational evidence showing the benefits of DOACs may be limited due to uncontrolled confounding, and the importance of weighing up the potential benefits to increased risk of bleeding at an individual level has also been highlighted [39].

In the current study, a decline in OAC use among people with VTE was observed, but this is likely because OAC use is only recommended for unprovoked VTE for 3–6 months [5]. The impact of extended use of DOACs in people with VTE living in LTCFs is unknown, and older adults with high bleeding risk, comorbidities and polypharmacy are underrepresented in VTE anticoagulation clinical trials [41]. However, in subgroup analyses of patients with frailty in clinical trials, extended treatment of VTE with DOACs reduced the risk of recurrent VTE with comparable or improved safety to standard therapy [42]. A higher incidence of VTE in people living in LTCFs compared to the older population living in the community has been previously observed [43]. For people with VTE in LTCFs, the net clinical benefit of reducing the risk of recurrent VTE versus increasing the risk of major bleeding needs to be considered when deciding whether individuals with prior unprovoked VTE should discontinue or continue OAC use [44].

In people with and without prior GI bleed, the results of the current study demonstrated similar prevalence use of OACs and a similar increase in use over time. Causes of prior upper GI tract bleeding, such as peptic ulcers, are usually preventable and therefore does not contraindicate the use of OACS [45]; however, untreatable causes of lower GI tract bleeding may contraindicate OACS. In an observational study of > 381 000 adults aged ≥ 75 years with non‐valvular AF at high risk of major bleeding, compared to warfarin, DOACs were associated with a lower risk of stroke; however, while apixaban and dabigatran were associated with a lower risk of major bleeding, rivaroxaban was associated with a higher risk [46]. Overall, careful selection of OAC type and consideration of bleeding aetiology are essential for optimising efficacy and safety in people with prior GI bleed in LTCFs.

## 5 Strengths and Limitations

Due to the longitudinal data capture in the ROSA National Historical Cohort, we were able to comprehensively examine changes in use of OACs by residents of LTCFs over time. Several limitations should be considered when interpreting the study results. OAC use was expected to be comprehensively captured for the four states examined, as all guideline‐recommended OACs are subsidised through the PBS. However, edoxaban and betrixaban are available for use in Australia but not subsidised by the Australian Government. We are also unable to examine medicines dispensed during hospitalisations.

Although national dispensing data were used, information on clinical indications, contraindications, dosage, adherence or adverse events was not available. OAC exposure was defined as at least one dispensing per calendar year; therefore, differences between short‐term, intermittent, or persistent OAC use were not examined, which may have led to overestimation of clinically meaningful exposure. This approach may also introduce survivorship bias. However, mortality rates were relatively stable across the study period (ranging from 23.7% to 25.0% between 2013 and 2018), suggesting that any such bias would be consistent over time and unlikely to substantially influence interpretation of temporal prescribing trends. Furthermore, the inclusion of new residents in the denominator, combined with a median (IQR) survival of 641 (218–1305) days from cohort entry, likely provides a reasonable representation of real‐world OAC prescribing patterns within LTCFs.

Health conditions such as AF and VTE were defined using a 12‐month look‐back period prior to study entry and were subsequently ascertained annually using the same 12‐month look‐back window until study end, which may have resulted in exposure misclassification. Cases diagnosed in primary care, those not requiring hospitalisation, or those with conditions diagnosed more than 12 months prior to cohort entry would not be captured, potentially leading to under‐ascertainment. We also did not further examine the temporal relationship between time of diagnoses and OAC use.

## 6 Conclusions

The use of OACs in people living in LTCFs has increased substantially since the introduction of DOACs, including in individuals with high‐risk comorbidities (prior GI bleed and dementia). However, uncertainty may remain among prescribing physicians when balancing the risks and benefits of OAC use to determine whether to prescribe or deprescribe OACs in older people with complex health care needs living in LTCFs. Individualised approaches are recommended when prescribing OACs, but further research of the benefits of OACs in LTCFs is critical to guide integrated approaches for the management of conditions which may require anticoagulation.

## Funding

The Registry of Senior Australians (ROSA) Research Centre is supported through the Australian Government Medical Research Future Fund (PHRDI000009) and its partners (South Australian Health and Medical Research Institute, Caring Futures Institute, College of Nursing and Health Sciences at Flinders University, ECH Inc., Silverchain and Bolton Clarke). M.C.I. is supported by a National Health and Medical Research Council (NHMRC) Investigator Grant (GNT119378). G.E.C. is supported by an NHMRC Investigator Grant (GNT2026400). J.K.S. is supported by a NHMRC Investigator Grant (GNT2016277).

## Ethics Statement

This study obtained ethics approvals from the University of South Australia Human Research Ethics Committee (Ref: 200489), the Australian Institute of Health and Welfare Ethics Committee (Ref: EO2022/4/1376), the South Australian Department for Health and Wellbeing Human Research Ethics Committee (Ref: HREC/18/SAH/90) for the inclusion of South Australia, Victorian and Queensland datasets and the New South Wales Population and Health Services Research Ethics Committee (Ref: 2019/ETH12028). These approvals are granted on the basis of a waiver of consent pursuant to s.95 of the Australian Privacy Act 1988 (Cth) and other state specific legislation.

## Conflicts of Interest

The authors declare no conflicts of interest.

## Supporting information


**Figure S1:** Yearly prevalence of oral anticoagulant use and prevalence ratio over the study period, for participants with and without dementia.
**Table S1:** International Classification of Diseases and Related Health Problems, Tenth Revision, Australian Modification (ICD‐10‐AM) codes used to identify gastrointestinal bleeding.
**Table S2:** Prevalence (95% confidence interval) of anticoagulants and antiplatelets by year adjusted by age and sex, by dementia status.
**Table S3:** Prevalence (95% confidence interval) of anticoagulants and antiplatelets by year adjusted by age and sex, by prior gastrointestinal bleed.
**Table S4:** Prevalence (95% confidence interval) of anticoagulants and antiplatelets by year adjusted by age and sex, for people with and without a history of atrial fibrillation.
**Table S5:** Prevalence (95% confidence interval) of anticoagulants and antiplatelets by year adjusted by age and sex, for people with and without a history of ischaemic stroke.
**Table S6:** Prevalence (95% confidence interval) of anticoagulants and antiplatelets by year adjusted by age and sex, for people with and without prior venous thromboembolism.
**Table S7:** Prevalence (95% confidence interval) of anticoagulants and antiplatelets by year adjusted by age and sex, for people with and without prior pulmonary embolism.
