# Supplementary material for: Oral Anticoagulant Use Among Older Adults in Long‐Term Care Facilities: Trends Over Time and Impact of High‐Risk Comorbidities
Source: Pharmacoepidemiol Drug Saf. 2026 Mar 30;35(4):e70356. doi: 10.1002/pds.70356 (PMC13036282; doi:10.1002/pds.70356)
Supplement: Supplementary file 1 — Figure S1: Yearly prevalence of oral anticoagulant use and prevalence ratio over the study period, for participants with and without dementia. Table S1: International Classification of Diseases and Related Health Problems, Tenth Revision, Australian Modification (ICD‐10‐AM) codes used to identify gastrointestinal bleeding. Table S2: Prevalence (95% confidence interval) of anticoagulants and antiplatelets by year adjusted by age and sex, by dementia status. Table S3: Prevalence (95% confidence interval) of anticoagulants and antiplatelets by year adjusted by age and sex, by prior gastrointestinal bleed. Table S4: Prevalence (95% confidence interval) of anticoagulants and antiplatelets by year adjusted by age and sex, for people with and without a history of atrial fibrillation. Table S5: Prevalence (95% confidence interval) of anticoagulants and antiplatelets by year adjusted by age and sex, for people with and without a history of ischaemic stroke. Table S6: Prevalence (95% confidence interval) of anticoagulants and antiplatelets by year adjusted by age and sex, for people with and without prior venous thromboembolism. Table S7: Prevalence (95% confidence interval) of anticoagulants and antiplatelets by year adjusted by age and sex, for people with and without prior pulmonary embolism. [file PDS-35-e70356-s001.docx]

**Supplementary Figure 1. Yearly prevalence of oral anticoagulant use and prevalence ratio over the study period, for participants with and without dementia.**

**
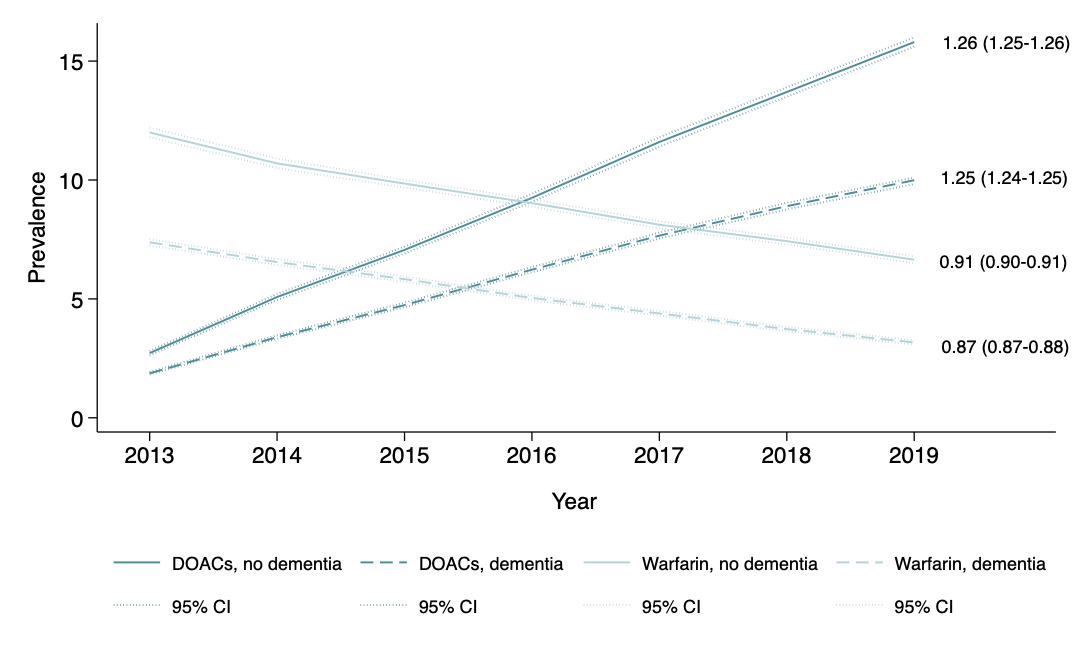
**

CI: confidence interval; DOAC: direct oral anticoagulant; OAC: oral anticoagulant.
Values displayed at the end of each line are adjusted prevalence ratios and 95% confidence intervals.

**Supplementary Table 1. International Classification of Diseases and Related Health Problems, Tenth Revision, Australian Modification (ICD-10-AM)** **codes used to identify gastrointestinal bleeding.**

| **ICD-10-AM code** | **Definition** |
| --- | --- |
| K25.0* | Gastric ulcer: acute with haemorrhage |
| K25.2 * | Gastric ulcer: acute with both haemorrhage and perforation |
| K25.4* | Gastric ulcer: chronic or unspecified with haemorrhage |
| K25.6* | Gastric ulcer: chronic or unspecified with both haemorrhage and perforation |
| K26.0* | Duodenal ulcer: acute with haemorrhage |
| K26.2 * | Duodenal ulcer: acute with both haemorrhage and perforation |
| K26.4 * | Duodenal ulcer: chronic or unspecified with haemorrhage |
| K26.6* | Duodenal ulcer: chronic or unspecified with both haemorrhage and perforation |
| K27.0* | Peptic ulcer, site unspecified: acute with haemorrhage |
| K27.2 * | Peptic ulcer, site unspecified: acute with both haemorrhage and perforation |
| K27.4* | Peptic ulcer, site unspecified: chronic or unspecified with haemorrhage |
| K27.6* | Peptic ulcer, site unspecified: chronic or unspecified with both haemorrhage and perforation |
| K28.0* | Gastrojejunal ulcer: acute with haemorrhage |
| K28.2 * | Gastrojejunal ulcer: acute with both haemorrhage and perforation |
| K28.4* | Gastrojejunal ulcer: chronic or unspecified with haemorrhage |
| K28.6* | Gastrojejunal ulcer: chronic or unspecified with both haemorrhage and perforation |
| K29.0 | Acute haemorrhagic gastritis |
| K29.31 | Chronic superficial gastritis with bleeding |
| K29.41 | Chronic atrophic gastritis with bleeding |
| K29.51 | Unspecified chronic gastritis with bleeding |
| K29.61 | Other gastritis with bleeding |
| K29.71 | Gastritis, unspecified, with bleeding |
| K29.81 | Duodenitis with bleeding |
| K29.91 | Gastroduodenitis |
| K31.82 | Dieulafoy lesion (hemorrhagic) of stomach and duodenum |
| K92.2 | Gastrointestinal haemorrhage, unspecified |

**Supplementary Table 2. Prevalence (95% confidence interval) of anticoagulants and antiplatelets by year adjusted by age and sex, by dementia status.**

|  | **2013** | **2014** | **2015** | **2016** | **2017** | **2018** | **2019** | **Adjusted Prevalence Ratio** |
| --- | --- | --- | --- | --- | --- | --- | --- | --- |
| **Dementia (N=292,357)** | N=122,992 | N=122,671 | N=122,247 | N=121,438 | N=120,948 | N=118,220 | N=116,435 |  |
| Any OAC | 9.93 (9.79-10.1) | 10.3 (10.2-10.4) | 10.7 (10.6-10.9) | 11.2 (11.1-11.4) | 11.8 (11.7-12.0) | 12.3 (12.1-12.4) | 12.8 (12.6-12.9) | 1.04 (1.04-1.05) |
| Warfarin | 7.38 (7.25-7.51) | 6.55 (6.43-6.67) | 5.83 (5.72-5.94) | 5.04 (4.94-5.14) | 4.39 (4.30-4.49) | 3.73 (3.64-3.82) | 3.17 (3.08-3.26) | 0.87 (0.87-0.88) |
| DOAC | 1.87 (1.82-1.93) | 3.39 (3.32-3.47) | 4.74 (4.65-4.84) | 6.23 (6.12-6.34) | 7.66 (7.54-7.79) | 8.90 (8.76-9.04) | 9.99 (9.83-10.1) | 1.25 (1.24-1.25) |
| Rivaroxaban | 0.89 (0.85-0.94) | 1.69 (1.63-1.74) | 2.25 (2.18-2.32) | 2.74 (2.66-2.82) | 3.06 (2.98-3.14) | 3.23 (3.14-3.32) | 3.40 (3.30-3.49) | 1.17 (1.16-1.18) |
| Dabigatran | 0.43 (0.40-0.46) | 0.66 (0.62-0.70) | 0.69 (0.65-0.72) | 0.76 (0.72-0.80) | 0.86 (0.81-0.90) | 0.88 (0.84-0.93) | 0.84 (0.79-0.89) | 1.09 (1.08-1.10) |
| Apixaban | 0.44 (0.42-0.46) | 0.96 (0.93-1.00) | 1.79 (1.73-1.84) | 2.81 (2.73-2.88) | 3.91 (3.82-4.00) | 5.03 (4.93-5.14) | 6.04 (5.93-6.17) | 1.39 (1.38-1.40) |
| Heparin | 3.45 (3.35-3.56) | 3.34 (3.24-3.44) | 3.27 (3.17-3.37) | 3.20 (3.10-3.30) | 3.17 (3.08-3.27) | 2.83 (2.74-2.93) | 2.64 (2.55-2.73) | 0.96 (0.95-0.97) |
| Antiplatelets | 41.2 (40.9-41.4) | 38.3 (38.0-38.5) | 35.1 (34.8-35.3) | 21.0 (20.8-21.2) | 17.1 (16.9-17.3) | 15.3 (15.1-15.5) | 14.0 (13.8-14.1) | 0.82 (0.81-0.82) |
| Antiplatelets (no aspirin) | 11.2 (11.1-11.4) | 10.6 (10.4-10.7) | 9.94 (9.80-10.1) | 10.2 (10.0-10.3) | 9.45 (9.32-9.59) | 8.76 (8.62-8.90) | 8.13 (7.99-8.27) | 0.95 (0.95-0.95) |
| Aspirin | 29.8 (29.5-30.0) | 27.7 (27.4-27.9) | 25.2 (25.0-25.5) | 11.0 (10.9-11.2) | 7.84 (7.70-7.99) | 6.70 (6.56-6.84) | 5.96 (5.82-6.09) | 0.74 (0.74-0.74) |
| **No dementia (N=208,526)** | N=67,871 | N=70,390 | N=73,216 | N=76,094 | N=79,627 | N=82,295 | N=86,849 |  |
| Any OAC | 15.7 (15.4-15.9) | 16.3 (16.1-16.5) | 17.1 (16.9-17.3) | 18.1 (17.9-18.3) | 19.3 (19.1-19.5) | 20.5 (20.3-20.8) | 21.7 (21.4-21.9) | 1.06 (1.06-1.06) |
| Warfarin | 12.0 (11.8-12.2) | 10.7 (10.5-10.9) | 9.85 (9.68-10.0) | 9.03 (8.87-9.19) | 8.12 (7.97-8.27) | 7.43 (7.29-7.58) | 6.65 (6.51-6.79) | 0.91 (0.90-0.91) |
| DOAC | 2.72 (2.62-2.81) | 5.08 (4.95-5.21) | 7.06 (6.91-7.20) | 9.25 (9.08-9.42) | 11.6 (11.4-11.8) | 13.7 (13.5-13.9) | 15.8 (15.6-16.0) | 1.26 (1.25-1.26) |
| Rivaroxaban | 1.31 (1.24-1.38) | 2.43 (2.33-2.52) | 3.09 (2.99-3.19) | 3.67 (3.56-3.78) | 4.03 (3.92-4.15) | 4.33 (4.21-4.45) | 4.52 (4.39-4.65) | 1.15 (1.15-1.16) |
| Dabigatran | 0.59 (0.54-0.64) | 0.92 (0.86-0.98) | 0.90 (0.85-0.96) | 0.99 (0.93-1.04) | 1.18 (1.12-1.24) | 1.29 (1.22-1.35) | 1.32 (1.25-1.39) | 1.11 (1.10-1.13) |
| Apixaban | 0.57 (0.54-0.60) | 1.58 (1.52-1.65) | 2.98 (2.88-3.08) | 4.69 (4.56-4.81) | 6.64 (6.50-6.79) | 8.53 (8.36-8.69) | 10.5 (10.3-10.7) | 1.40 (1.39-1.41) |
| Heparin | 5.47 (5.31-5.64) | 5.48 (5.31-5.64) | 5.37 (5.21-5.53) | 5.08 (4.92-5.22) | 4.91 (4.76-5.05) | 4.57 (4.43-4.71) | 4.06 (3.93-4.19) | 0.95 (0.95-0.96) |
| Antiplatelets | 42.0 (41.6-42.3) | 39.0 (38.7-39.4) | 36.6 (36.3-36.9) | 23.2 (22.9-23.5) | 19.6 (19.4-19.9) | 18.2 (17.8-18.5) | 16.9 (16.6-17.1) | 0.84 (0.84-0.84) |
| Antiplatelets (no aspirin) | 12.3 (12.1-12.6) | 11.6 (11.4-11.9) | 11.1 (10.9-11.3) | 11.9 (11.7-12.1) | 11.5 (11.3-11.7) | 11.0 (10.8-11.2) | 10.4 (10.2-10.6) | 0.98 (0.97-0.98) |
| Aspirin | 29.5 (29.2-29.9) | 27.5 (27.2-27.8) | 25.7 (25.4-26.0) | 11.5 (11.3-11.7) | 8.35 (8.16-8.53) | 7.46 (7.29-7.64) | 6.67 (6.51-6.84) | 0.75 (0.75-0.76) |

DOAC: direct oral anticoagulant; OAC: oral anticoagulant.

**Supplementary Table 3. Prevalence (95% confidence interval) of anticoagulants and antiplatelets by year adjusted by age and sex, by prior gastrointestinal bleed.**

|  | **2013** | **2014** | **2015** | **2016** | **2017** | **2018** | **2019** | **Adjusted Prevalence Ratio** |
| --- | --- | --- | --- | --- | --- | --- | --- | --- |
| **GI bleed (N=14,628)** | N=2,587 | N=3639 | N=4424 | N=4930 | N=5409 | N=5665 | N=5994 |  |
| Any OAC | 12.9 (11.8-13.9) | 14.1 (13.2-15.0) | 14.3 (13.5-15.1) | 14.5 (13.7-15.2) | 15.9 (15.1-16.7) | 17.0 (16.2-17.8) | 17.1 (16.2-17.9) | 1.05 (1.03-1.06) |
| Warfarin | 10.5 (9.46-11.5) | 9.97 (9.19-10.7) | 8.71 (8.04-9.37) | 7.50 (6.92-8.07) | 6.89 (6.34-7.43) | 6.73 (6.19-7.27) | 5.64 (5.11-6.16) | 0.90 (0.88-0.92) |
| DOAC | 1.99 (1.56-2.42) | 4.09 (3.57-4.61) | 5.56 (5.03-6.10) | 7.18 (6.59-7.78) | 9.36 (8.71-10.0) | 11.1 (10.4-11.8) | 12.2 (11.4-12.9) | 1.24 (1.21-1.27) |
| Rivaroxaban | 1.22 (0.85-1.59) | 2.06 (1.67-2.46) | 2.62 (2.23-3.00) | 2.72 (2.35-3.10) | 3.07 (2.69-3.46) | 3.33 (2.91-3.75) | 3.32 (2.89-3.76) | 1.11 (1.07-1.15) |
| Dabigatran | 0.42 (0.24-0.61) | 0.73 (0.51-0.95) | 0.50 (0.34-0.67) | 0.79 (0.57-1.00) | 1.06 (0.83-1.28) | 1.27 (1.03-1.52) | 1.27 (1.00-1.53) | 1.18 (1.11-1.26) |
| Apixaban | 0.33 (0.17-0.49) | 1.33 (1.04-1.62) | 2.44 (2.08-2.80) | 3.74 (3.30-4.17) | 5.35 (4.84-5.86) | 6.75 (6.17-7.34) | 7.87 (7.22-8.53) | 1.36 (1.32-1.40) |
| Heparin | 4.73 (3.94-5.53) | 5.38 (4.68-6.09) | 4.57 (3.98-5.17) | 4.73 (4.15-5.30) | 4.33 (3.81-4.86) | 4.13 (3.62-4.64) | 3.39 (2.93-3.84) | 0.94 (0.92-0.97) |
| Antiplatelets | 34.4 (32.7-36.1) | 33.2 (31.8-32.2) | 31.0 (29.7-32.2) | 19.9 (18.9-20.9) | 16.3 (15.5-17.2) | 15.5 (14.6-16.3) | 14.4 (13.5-15.2) | 0.84 (0.83-0.85) |
| Antiplatelets (no aspirin) | 11.2 (10.1-12.3) | 11.2 (10.3-12.1) | 10.2 (9.46-11.0) | 10.9 (9.42-10.8) | 10.1 (9.42-10.8) | 9.37 (8.73-10.0) | 8.66 (8.00-9.32) | 0.96 (0.94-0.98) |
| Aspirin | 23.0 (21.5-24.5) | 22.2 (20.9-23.5) | 20.8 (19.7-22.0) | 9.17 (8.40-9.93) | 6.46 (5.82-7.10) | 6.19 (5.58-6.80) | 5.86 (5.27-6.46) | 0.75 (0.74-0.77) |
| **No GI bleed (N=486,255)** | N=185,763 | N=187,102 | N=188,893 | N=190,715 | N=193654 | N=193976 | N=197290 |  |
| Any OAC | 12.1 (12.0-12.2) | 12.6 (12.5-12.8) | 13.3 (13.1-13.4) | 14.0 (13.9-14.1) | 14.9 (14.7-15.0) | 15.7 (15.5-15.8) | 16.5 (16.4-16.7) | 1.05 (1.05-1.06) |
| Warfarin | 9.11 (9.00-9.23) | 8.15 (8.05-8.25) | 7.41 (7.32-7.51) | 6.63 (6.55-6.72) | 5.89 (5.81-5.97) | 5.22 (5.14-5.30) | 4.61 (4.53-4.69) | 0.89 (0.89-0.90) |
| DOAC | 2.22 (2.17-2.27) | 4.06 (3.99-4.13) | 5.65 (5.57-5.74) | 7.43 (7.34-7.53) | 9.23 (9.13-9.34) | 10.9 (10.8-11.0) | 12.5 (12.3-12.6 | 1.26 (1.25-1.26) |
| Rivaroxaban | 1.05 (1.01-1.09) | 1.97 (1.92-2.02) | 2.58 (2.52-2.63) | 3.11 (3.05-3.18) | 3.45 (3.38-3.52) | 3.69 (3.62-3.77) | 3.90 (3.82-3.97) | 1.17 (1.16-1.17) |
| Dabigatran | 0.49 (0.46-0.52) | 0.76 (0.73-0.81) | 0.78 (0.74-0.81) | 0.85 (0.81-0.88) | 0.98 (0.94-1.02) | 1.04 (1.00-1.08) | 1.04 (1.00-1.08) | 1.10 (1.09-1.11) |
| Apixaban | 0.51 (0.49-0.53) | 1.21 (1.18-1.25) | 2.25 (2.20-2.30) | 3.55 (3.48-3.61) | 5.00 (4.92-5.08) | 6.47 (6.37-6.56) | 7.93 (7.82-8.04) | 1.40 (1.40-1.41) |
| Heparin | 4.18 (4.09-4.27) | 4.09 (4.00-4.18) | 4.05 (3.96-4.14) | 3.90 (3.81-3.98) | 3.84 (3.75-3.92) | 3.51 (3.43-3.59) | 3.24 (3.16-3.31) | 0.96 (.096-0.97) |
| Antiplatelets | 41.5 (41.3-41.7) | 38.6 (38.4-38.8) | 35.7 (35.5-35.9) | 21.9 (21.8-22.1) | 18.2 (18.0-18.3) | 16.5 (16.4-16.7) | 15.3 (15.1-15.4) | 0.83 (0.83-0.83) |
| Antiplatelets (no aspirin) | 11.7 (11.6-11.8) | 11.0 (10.9-11.1) | 10.4 (10.3-10.5) | 10.8 (10.7-11.0) | 10.3 (10.2-10.4) | 9.68 (9.57-9.80) | 9.10 (8.99-9.22) | 0.96 (0.96-0.97) |
| Aspirin | 29.7 (29.5-29.9) | 27.6 (27.4-27.8) | 25.5 (25.3-25.6) | 11.3 (11.1-11.4) | 8.08 (7.96-8.19) | 7.03 (6.92-7.14) | 6.28 (6.18-6.39) | 0.75 (0.74-0.75) |

DOAC: direct oral anticoagulant; OAC: oral anticoagulant.

**Supplementary Table 4. Prevalence (95% confidence interval) of anticoagulants and antiplatelets by year adjusted by age and sex, for people with and without a history of atrial fibrillation.**

| ` | **2013** | **2014** | **2015** | **2016** | **2017** | **2018** | **2019** | **Adjusted Prevalence Ratio** |
| --- | --- | --- | --- | --- | --- | --- | --- | --- |
| **Atrial fibrillation (N=60,018)** | N=11,885 | N=15,926 | N=18,590 | N=20,422 | N=22,277 | N=23,151 | N=23,998 |  |
| Any OAC | 35.2 (34.5-35.9) | 36.4 (35.9-37.0) | 37.7 (37.2-38.2) | 39.1 (38.6-39.7) | 40.7 (40.2-41.2) | 41.9 (41.4-42.4) | 42.9 (42.3-43.4) | 1.03 (1.03-1.04) |
| Warfarin | 25.1 (24.4-25.8) | 21.4 (20.8-21.9) | 18.8 (18.4-19.3) | 16.6 (16.2-17.0) | 14.7 (14.3-15.1) | 13.1 (12.7-13.4) | 11.4 (11.1-11.8) | 0.88 (0.87-0.89) |
| DOAC | 7.75 (7.40-8.09) | 14.1 (13.7-14.5) | 18.7 (18.2-19.1) | 23.0 (22.5-23.4) | 26.7 (26.3-27.2) | 29.9 (29.4-30.4) | 32.6 (32.1-33.2) | 1.27 (1.26-1.28) |
| Rivaroxaban | 3.41 (3.15-3.67) | 5.89 (5.59-6.19) | 7.03 (6.74-7.33) | 7.98 (7.68-8.27) | 8.13 (7.84-8.42) | 8.32 (8.01-8.62) | 8.41 (8.08-8.74) | 1.08 (1.07-1.09) |
| Dabigatran | 1.29 (1.10-1.47) | 2.09 (1.89-2.29) | 2.17 (1.99-2.34) | 2.25 (2.08-2.41) | 2.77 (2.59-2.95) | 2.82 (2.63-3.01) | 2.75 (2.55-2.96) | 1.09 (1.06-1.11) |
| Apixaban | 2.47 (2.31-2.62) | 5.64 (5.39-5.89) | 9.22 (8.92-9.53) | 12.8 (12.5-13.2) | 16.2 (15.9-16.6) | 19.6 (19.1-20.0) | 22.5 (22.0-23.0) | 1.27 (1.27-1.28) |
| Heparin | 5.89 (5.49-6.31) | 5.21 (4.87-5.54) | 4.82 (4.52-5.12) | 4.18 (3.92-4.45) | 4.11 (3.85-4.36) | 3.47 (3.24-3.70) | 3.08 (2.87-3.30) | 0.90 (0.89-0.91) |
| Antiplatelets | 46.8 (46.0-47.7) | 42.5 (41.8-43.3) | 37.9 (37.2-38.5) | 21.6 (21.1-22.1) | 17.0 (16.6-17.5) | 14.5 (14.1-14.9) | 12.7 (12.3-13.1) | 0.78 (0.78-0.79) |
| Antiplatelets (no aspirin) | 11.3 (10.7-11.8) | 10.4 (10.0-10.8) | 9.56 (9.20-9.91) | 9.98 (9.64-10.3) | 9.33 (9.02-9.64) | 8.20 (7.90-8.50) | 7.21 (6.92-7.51) | 0.94 (0.93-0.94) |
| Aspirin | 35.6 (34.8-36.4) | 32.2 (31.5-32.9) | 28.4 (27.8-29.1) | 11.9 (11.4-12.3) | 8.01 (7.67-8.35) | 6.54 (6.23-6.84) | 5.56 (5.28-5.85) | 0.70 (0.70-0.71) |
| **No atrial fibrillation (N=440,865)** | N=171,891 | N=170,768 | N=171,080 | N=171,969 | N=174,206 | N=175,004 | N=179,286 |  |
| Any OAC | 9.62 (9.51-9.74) | 9.84 (9.73-9.95) | 10.2 (10.1-10.3) | 10.6 (10.5-10.8) | 11.2 (11.1-11.3) | 11.8 (11.7-11.9) | 12.7 (12.6-12.9) | 1.05 (1.05-1.05) |
| Warfarin | 7.37 (7.26-7.47) | 6.53 (6.44-6.63) | 5.91 (5.82-6.00) | 5.27 (5.19-5.36) | 4.65 (4.58-4.73) | 4.11 (4.03-4.18) | 3.65 (3.58-3.73) | 0.89 (0.89-0.89) |
| DOAC | 1.65 (1.61-1.69) | 2.94 (2.88-3.00) | 4.08 (4.01-4.15) | 5.39 (5.30-5.47) | 6.72 (6.62-6.82) | 8.00 (7.89-8.10) | 9.51 (9.39-9.64) | 1.27 (1.26-1.27) |
| Rivaroxaban | 0.83 (0.80-0.86) | 1.52 (1.47-1.57) | 2.00 (1.95-2.05) | 2.41 (2.35-2.47) | 2.74 (2.68-2.81) | 2.96 (2.89-3.02) | 3.20 (3.13-3.28) | 1.18 (1.17-1.19) |
| Dabigatran | 0.39 (0.37-0.41) | 0.59 (0.56-0.62) | 0.59 (0.56-0.62) | 0.66 (0.63-0.69) | 0.73 (0.69-0.76) | 0.78 (0.74-0.81) | 0.80 (0.76-0.84) | 1.10 (1.09-1.11) |
| Apixaban | 0.31 (0.30-0.33) | 0.76 (0.72-0.77) | 1.44 (1.39-1.48) | 2.36 (2.31-2.42) | 3.40 (3.33-3.47) | 4.47 (4.39-4.56) | 5.81 (5.71-5.91) | 1.44 (1.43-1.45) |
| Heparin | 3.98 (3.89-4.07) | 3.96 (3.87-4.04) | 3.94 (3.85-4.03) | 3.84 (3.75-3.93) | 3.77 (3.68-3.86) | 3.49 (3.41-3.58) | 3.26 (3.18-3.34) | 0.97 (0.96-0.97) |
| Antiplatelets | 41.0 (40.8-41.3) | 38.1 (37.9-38.3) | 35.3 (35.1-35.5) | 21.8 (21.7-22.0) | 18.2 (18.0-18.4) | 16.7 (16.5-16.8) | 15.5 (15.4-15.7) | 0.83 (0.83-0.83) |
| Antiplatelets (no aspirin) | 11.8 (11.7-11.9) | 11.1 (11.0-11.2) | 10.5 (10.4-10.6) | 10.9 (10.8-11.1) | 10.4 (10.2-10.5) | 9.86 (9.74-9.98) | 9.34 (9.22-9.45) | 0.97 (0.96-0.97) |
| Aspirin | 29.1 (28.9-29.3) | 27.0 (26.8-27.2) | 24.9 (24.7-25.1) | 11.1 (10.9-11.2) | 7.98 (7.86-8.10) | 7.00 (6.89-7.11) | 6.33 (6.22-6.44) | 0.75 (0.75-0.75) |

DOAC: direct oral anticoagulant; OAC: oral anticoagulant.

**Supplementary Table 5. Prevalence (95% confidence interval) of anticoagulants and antiplatelets by year adjusted by age and sex, for people with and without a history of ischaemic stroke.**

|  | **2013** | **2014** | **2015** | **2016** | **2017** | **2018** | **2019** | **Adjusted Prevalence Ratio** |
| --- | --- | --- | --- | --- | --- | --- | --- | --- |
| **Ischaemic stroke (N=17818)** | N=3,412 | N=4,660 | N=5,655 | N=6,494 | N=7,303 | N=7,827 | N=8,445 |  |
| Any OAC | 31.2 (30.1-32.4) | 31.6 (30.6-32.6) | 32.2 (31.3-33.1) | 31.8 (31.0-32.7) | 32.0 (31.1-32.8) | 32.2 (31.4-33.1) | 32.1 (31.2-33.0) | 1.00 (1.00-1.01)* |
| Warfarin | 18.4 (17.2-19.6) | 15.5 (14.6-16.4) | 13.7 (13.0-14.5) | 11.9 (11.3-12.5) | 10.1 (9.57-10.7) | 9.12 (8.58-9.66) | 8.15 (7.61-8.68) | 0.87 (0.86-0.89) |
| DOAC | 8.86 (8.23-9.48) | 14.7 (13.9-15.4) | 17.9 (17.1-18.6) | 20.0 (19.3-20.8) | 22.4 (21.6-23.1) | 24.1 (23.3-24.9) | 25.2 (24.3-26.0) | 1.12 (1.11-1.13) |
| Rivaroxaban | 2.50 (2.04-2.95) | 4.38 (3.88-4.88) | 5.13 (4.67-5.59) | 5.28 (4.84-5.72) | 5.32 (4.90-5.75) | 5.06 (4.64-5.48) | 5.01 (4.58-5.45) | 1.04 (1.01-1.07) |
| Dabigatran | 1.15 (0.84-1.46) | 1.98 (1.64-2.32) | 2.02 (1.74-2.31) | 2.02 (1.75-2.29) | 2.43 (2.14-2.72) | 2.47 (2.17-2.77) | 2.26 (1.97-2.55) | 1.06 (1.02-1.10) |
| Apixaban | 4.25 (3.93-4.58) | 7.57 (7.10-8.05) | 10.4 (9.85-10.9) | 12.7 (12.1-13.3) | 14.9 (14.3-15.5) | 17.2 (16.5-17.8) | 18.7 (17.9-19.4) | 1.18 (1.17-1.20) |
| Heparin | 5.90 (5.13-6.66) | 5.37 (4.74-6.00) | 4.58 (4.06-5.11) | 4.55 (4.06-5.05) | 3.78 (3.36-4.21) | 3.32 (2.93-3.71) | 3.14 (2.77-3.51) | 0.90 (0.88-0.92) |
| Antiplatelets | 61.3 (59.7-62.8) | 58.5 (57.2-59.8) | 54.5 (53.3-55.7) | 39.2 (38.2-40.2) | 34.1 (33.1-35.0) | 31.2 (30.2-32.1) | 29.0 (28.1-30.0) | 0.87 (0.86-0.87) |
| Antiplatelets (no aspirin) | 26.8 (25.5-28.1) | 25.3 (24.2-26.3) | 23.9 (23.0-24.8) | 25.0 (24.2-25.9) | 23.7 (22.9-24.5) | 22.2 (21.5-23.0) | 20.9 (20.0-21.7) | 0.96 (0.95-0.97) |
| Aspirin | 34.9 (33.4-36.4) | 33.5 (32.2-34.8) | 31.3 (30.1-32.4) | 14.3 (13.5-15.1) | 10.4 (9.78-11.1) | 9.03 (8.43-9.64) | 8.11 (7.54-8.69) | 0.75 (0.74-0.76) |
| **No ischaemic stroke (N=483,065)** | N=186,123 | N=187,128 | N=188,547 | N=189,888 | N=192,295 | N=192,122 | N=194,839 |  |
| Any OAC | 11.8 (11.7-11.9) | 12.2 (12.1-12.3) | 12.7 (12.6-12.9) | 13.4 (13.3-13.6) | 14.3 (14.2-14.4) | 15.0 (14.9-15.2) | 15.9 (15.7-16.0) | 1.05 (1.05-1.05) |
| Warfarin | 8.96 (8.85-9.08) | 8.00 (7.90-8.10) | 7.25 (7.15-7.34) | 6.49 (6.40-6.57) | 5.77 (5.69-5.86) | 5.11 (5.03-5.20) | 4.49 (4.41-4.57) | 0.89 (0.89-0.90) |
| DOAC | 2.08 (2.03-2.13) | 3.81 (3.74-3.88) | 5.32 (5.24-5.40) | 7.03 (6.94-7.12) | 8.77 (8.67-8.88) | 10.4 (10.2-10.5) | 11.9 (11.8-12.0) | 1.26 (1.26-1.26) |
| Rivaroxaban | 1.02 (0.98-1.05) | 1.91 (1.86-1.96) | 2.50 (2.44-2.55) | 3.03 (2.97-3.09) | 3.38 (3.32-3.45) | 3.63 (3.56-3.70) | 3.84 (3.76-3.91) | 1.17 (1.16-1.17) |
| Dabigatran | 0.48 (0.46-0.51) | 0.73 (0.70-0.77) | 0.74 (0.71-0.77) | 0.81 (0.78-0.85) | 0.94 (0.90-0.97) | 0.99 (0.95-1.03) | 0.99 (0.95-1.03) | 1.10 (1.09-1.11) |
| Apixaban | 0.44 (0.42-0.45) | 1.06 (1.03-1.10) | 2.03 (1.98-2.08) | 3.25 (3.19-3.32) | 4.64 (4.56-4.72) | 6.03 (5.94-6.12) | 7.45 (7.34-7.56) | 1.42 (1.41-1.42) |
| Heparin | 4.16 (4.07-4.25) | 4.10 (4.01-4.19) | 4.05 (3.96-4.14) | 3.91 (3.82-4.00) | 3.87 (3.78-3.95) | 3.55 (3.47-3.63) | 3.25 (3.17-3.32) | 0.96 (0.96-0.97) |
| Antiplatelets | 41.0 (40.8-41.3) | 38.0 (37.8-38.2) | 35.1 (34.9-35.3) | 21.3 (21.1-21.5) | 17.5 (17.4-17.7) | 15.9 (15.7-16.0) | 14.6 (14.5-14.8) | 0.82 (0.82-0.82) |
| Antiplatelets (no aspirin) | 11.4 (11.3-11.5) | 10.7 (10.5-10.8) | 10.0 (9.91-10.1) | 10.4 (10.3-10.5) | 9.78 (9.67-9.89) | 9.14 (9.03-9.24) | 8.55 (8.44-8.66) | 0.96 (0.96-0.96) |
| Aspirin | 29.5 (29.3-29.7) | 27.4 (27.2-27.6) | 25.2 (25.0-25.4) | 11.1 (11.0-11.2) | 7.93 (7.81-8.05) | 6.90 (6.79-7.01) | 6.18 (6.07-6.29 | 0.74 (0.74-0.75) |

*Not statistically significant.

DOAC: direct oral anticoagulant; OAC: oral anticoagulant.

**Supplementary Table 6. Prevalence (95% confidence interval) of anticoagulants and antiplatelets by year adjusted by age and sex, for people with and without prior venous thromboembolism.**

|  | **2013** | **2014** | **2015** | **2016** | **2017** | **2018** | **2019** | **Adjusted Prevalence Ratio** |
| --- | --- | --- | --- | --- | --- | --- | --- | --- |
| **VTE (N=11,771)** | N=1,979 | N=2,810 | N=3,539 | N=3,933 | N=4,469 | N=4,812 | N=5,209 |  |
| Any OAC | 55.8 (53.7-57.9) | 51.1 (49.4-52.8) | 48.1 (46.7-49.5) | 45.5 (44.2-46.8) | 44.0 (42.8-45.2) | 43.3 (42.1-44.6) | 41.6 (40.3-42.8) | 0.96 (0.95-0.96) |
| Warfarin | 45.8 (43.5-48.1) | 35.6 (33.9-37.3) | 28.6 (27.3-29.9) | 22.3 (21.2-23.5) | 17.1 (16.2-18.1) | 14.0 (13.1-14.9) | 11.0 (10.2-11.9) | 0.79 (0.78-0.80) |
| DOAC | 8.88 (7.99-9.77) | 14.0 (13.0-14.9) | 19.4 (18.4-20.5) | 23.7 (22.6-24.8) | 27.4 (26.3-28.5) | 30.8 (29.6-31.9) | 32.2 (31.0-33.3) | 1.16 (1.15-1.18) |
| Rivaroxaban | 7.01 (6.16-7.86) | 10.9 (10.0-11.8) | 13.8 (12.9-14.7) | 14.8 (13.9-15.7) | 14.3 (13.4-15.1) | 13.5 (12.7-14.4) | 12.2 (11.2-13.0) | 1.02 (1.01-1.04) |
| Dabigatran | 0.44 (0.18-0.69) | 0.44 (0.22-0.67) | 0.62 (0.37-0.86) | 0.65 (0.42-0.88) | 1.10 (0.83-1.37) | 1.22 (0.94-1.50) | 0.98 (0.72-1.24) | 1.16 (1.06-1.26) |
| Apixaban | 0.68 (0.54-0.82) | 1.74 (1.46-2.02) | 4.19 (3.73-4.65) | 7.86 (7.21-8.50) | 12.3 (11.5-13.1) | 17.1 (16.2-18.0) | 20.1 (19.1-21.1) | 1.44 (1.41-1.47) |
| Heparin | 19.6 (18.0-21.2) | 17.0 (15.7-18.2) | 14.5 (13.4-15.5) | 12.0 (11.1-12.9) | 10.8 (10.0-11.7) | 10.1 (9.33-10.9) | 7.95 (7.25-8.64) | 0.87 (0.85-0.88) |
| Antiplatelets | 29.5 (27.6-31.4) | 27.3 (25.8-28.8) | 25.9 (24.6-27.2) | 15.5 (14.5-16.5) | 12.0 (11.1-12.8) | 11.0 (11.2-11.8) | 10.5 (9.71-11.3) | 0.82 (0.80-0.83) |
| Antiplatelets (no aspirin) | 6.94 (5.90-7.98) | 6.48 (5.67-7.30) | 6.72 (6.02-7.41) | 7.06 (6.39-7.74) | 6.56 (5.95-7.17) | 6.24 (5.66-6.82) | 5.86 (5.27-6.82) | 0.94 (0.93-0.94) |
| Aspirin | 22.3 (20.6-24.1) | 20.7 (19.3-22.1) | 19.2 (17.9-20.4) | 8.59 (7.76-9.43) | 5.60 (4.94-6.25) | 4.98 (4.38-5.58) | 4.75 (4.18-5.32) | 0.73 (0.71-0.75) |
| **No VTE (N=489,112)** | N=187,260 | N=188,705 | N=190,441 | N=192,184 | N=194,947 | N=195,020 | N=198,075 |  |
| Any OAC | 11.7 (11.6-11.8) | 12.2 (12.1-12.3) | 12.7 (12.6-12.8) | 13.4 (13.3-13.5) | 14.2 (14.0-14.3) | 14.8 (14.7-15.0) | 15.7 (15.6-15.9) | 1.05 (1.05-1.05) |
| Warfarin | 8.76 (8.65-8.87) | 7.80 (7.70-7.90) | 7.06 (6.97-7.15) | 6.32 (6.24-6.41) | 5.63 (5.55-5.71) | 4.98 (4.91-5.06) | 4.42 (4.34-4.94) | 0.89 (0.89-0.90) |
| DOAC | 2.19 (2.14-2.23) | 3.95 (3.89-4.02) | 5.46 (5.38-5.54) | 7.12 (7.03-7.21) | 8.79 (8.69-8.89) | 10.3 (10.1-10.4) | 11.8 (11.7-12.0) | 1.25 (1.25-1.25) |
| Rivaroxaban | 1.01 (0.97-1.04) | 1.84 (1.79-1.89) | 2.38 (2.32-2.43) | 2.85 (2.79-2.92) | 3.16 (3.10-3.22) | 3.37 (3.30-3.44) | 3.61 (3.54-3.69) | 1.16 (1.16-1.17) |
| Dabigatran | 0.50 (0.47-0.53) | 0.77 (0.74-0.80) | 0.78 (0.75-0.81) | 0.86 (0.82-0.89) | 0.98 (0.95-1.02) | 1.04 (1.00-1.08) | 1.04 (1.00-1.08) | 1.10 (1.09-1.11) |
| Apixaban | 0.52 (0.50-0.54) | 1.23 (1.20-1.27) | 2.26 (2.21-2.31) | 3.49 (3.42-3.55) | 4.83 (4.75-4.91) | 6.14 (6.05-6.23) | 7.57 (7.46-7.67) | 1.39 (1.38-1.39) |
| Heparin | 3.91 (3.83-4.00) | 3.84 (3.76-3.92) | 3.80 (3.72-3.88) | 3.69 (3.56-3.72) | 3.64 (3.56-3.72) | 3.31 (3.23-3.38) | 3.12 (3.04-3.19) | 0.96 (0.96-0.97) |
| Antiplatelets | 41.7 (41.4-41.9) | 38.8 (38.6-39.0) | 35.9 (35.7-36.1) | 22.0 (21.9-22.2) | 18.3 (18.1-18.5) | 16.7 (16.5-16.8) | 15.4 (15.2-15.5) | 0.83 (0.83-0.83) |
| Antiplatelets (no aspirin) | 11.8 (11.7-11.9) | 11.1 (11.0-11.2) | 10.5 (10.4-10.6) | 10.9 (10.8-11.0) | 10.4 (10.3-10.5) | 9.78 (9.67-9.89) | 9.18 (9.07-9.30) | 0.96 (0.96-0.97) |
| Aspirin | 29.1 (28.9-29.3) | 27.0 (26.8-27.2) | 24.9 (24.7-25.1) | 11.1 (10.9-11.2) | 7.98 (7.86-8.10) | 7.00 (6.89-7.11) | 6.33 (6.22-6.44) | 0.75 (0.75-0.75) |

DOAC: direct oral anticoagulant; OAC: oral anticoagulant; VTE: venous thromboembolism.

**Supplementary Table 7. Prevalence (95% confidence interval) of anticoagulants and antiplatelets by year adjusted by age and sex, for people with and without prior pulmonary embolism.**

|  | **2013** | **2014** | **2015** | **2016** | **2017** | **2018** | **2019** | **Adjusted prevalence ratio** |
| --- | --- | --- | --- | --- | --- | --- | --- | --- |
| **PE (N=5,038)** | N=809 | N=1,167 | N=1,440 | N=1,618 | N=1,908 | N=2,061 | N=2,299 |  |
| Any OAC | 68.7 (65.3-72.0) | 62.5 (59.8-65.1) | 59.6 (57.4-61.8) | 56.5 (54.4-58.6) | 54.1 (52.2-56.1) | 54.0 (52.1-55.9) | 51.6 (49.7-53.5) | 0.96 (0.95-0.97) |
| Warfarin | 56.8 (53.0-60.7) | 43.2 (40.4-46.0) | 35.2 (33.0-37.4) | 27.5 (25.6-29.5) | 20.4 (18.8-22.0) | 17.1 (15.6-18.6) | 13.0 (11.6-14.3) | 0.78 (0.77-0.80) |
| DOAC | 12.2 (10.6-13.7) | 17.9 (16.4-19.4) | 23.9 (22.3-25.6) | 30.0 (28.2-31.7) | 34.7 (28.2-31.7) | 38.1 (36.3-39.9) | 39.6 (37.8-41.5) | 1.15 (1.14-1.17) |
| Rivaroxaban | 9.31 (7.92-10.7) | 13.5 (12.0-14.9) | 16.9 (15.3-18.4) | 18.7 (17.2-20.2) | 19.0 (17.5-20.4) | 18.2 (16.8-19.7) | 16.3 (14.9-17.7) | 1.03 (1.01-1.06) |
| Dabigatran | 0.65 (0.23-1.06) | 0.47 (0.18-0.75) | 0.71 (0.33-1.10) | 0.64 (0.27-1.01) | 0.95 (0.56-1.33) | 1.45 (0.99-1.92) | 0.94 (0.55-1.32) | 1.12 (1.00-1.26) |
| Apixaban | No dispensing | 2.58 (2.09-3.08) | 4.87 (4.18-5.56) | 10.0 (8.93-11.1) | 15.3 (14.0-16.6) | 20.2 (18.7-21.7) | 24.2 (22.6-25.9) | 1.44 (1.40-1.47) |
| Heparin | 19.1 (16.6-21.5) | 19.7 (17.6-21.7) | 16.5 (14.7-18.2) | 12.7 (11.3-14.2) | 11.7 (10.4-13.0) | 10.9 (9.67-12.1) | 8.27 (7.22-9.32) | 0.86 (0.84-0.89) |
| Antiplatelets | 22.6 (20.0-25.3) | 21.0 (18.9-23.1) | 19.7 (17.8-21.6) | 12.1 (10.7-13.6) | 9.70 (8.49-10.9) | 8.36 (7.29-9.44) | 8.16 (7.10-9.22) | 0.82 (0.80-0.84) |
| Antiplatelets (no aspirin) | 5.85 (4.35-7.35) | 5.42 (4.21-6.63) | 4.53 (3.58-5.48) | 5.06 (4.14-5.98) | 4.80 (3.97-5.64) | 4.77 (3.99-5.54) | 4.28 (3.52-5.05) | 0.96 (0.92-1.01) |
| Aspirin | 16.9 (14.5-19.3) | 15.6 (13.6-17.5) | 15.2 (13.4-16.9) | 7.15 (5.96-8.34) | 5.06 (4.12-6.01) | 3.69 (2.90-4.47) | 3.88 (3.11-4.64) | 0.74 (0.72-0.77) |
| **No PE (N=495,845)** | N=189,422 | N=191,306 | N=193,436 | N=195,322 | N=198,173 | N=198,160 | N=200,985 |  |
| Any OAC | 12.0 (11.9-12.1) | 12.5 (12.3-12.6) | 13.0 (12.9-13.1) | 13.7 (13.6-13.8) | 14.5 (14.4-14.6) | 15.2 (15.1-15.3) | 16.1 (15.9-16.2) | 1.05 (1.05-1.05) |
| Warfarin | 8.99 (8.88-9.10) | 8.02 (7.92-8.12) | 7.26 (7.17-7.35) | 6.49 (6.41-6.58) | 5.77 (5.69-5.86) | 5.11 (5.03-5.19) | 4.51 (4.43-4.59) | 0.89 (0.89-0.90) |
| DOAC | 2.20 (2.16-2.26) | 4.02 (3.95-4.09) | 5.57 (5.49-5.65) | 7.27 (7.18-7.36) | 9.00 (8.90-9.10) | 10.5 (10.4-10.7) | 12.1 (12.0-12.2) | 1.25 (1.25-1.26) |
| Rivaroxaban | 1.04 (1.00-1.07) | 1.92 (1.87-1.97) | 2.48 (2.43-2.54) | 2.97 (2.91-3.04) | 3.28 (3.22-3.35) | 3.49 (3.42-3.56) | 3.71 (3.63-3.78) | 1.16 (1.16-1.17) |
| Dabigatran | 0.50 (0.47-0.52) | 0.77 (0.74-0.80) | 0.78 (0.75-0.81) | 0.86 (0.82-0.89) | 0.99 (0.95-1.03) | 1.04 (1.00-1.08) | 1.04 (1.00-1.08) | 1.10 (1.09-1.11) |
| Apixaban | 0.51 (0.50-0.53) | 1.22 (1.19-1.26) | 2.27 (2.22-2.32) | 3.52 (3.45-3.58) | 4.92 (4.84-5.00) | 6.30 (6.21-6.40) | 7.72 (7.61-7.82) | 1.39 (1.39-1.40) |
| Heparin | 4.09 (4.00-4.18) | 4.01 (3.92-4.09) | 3.95 (3.87-4.04) | 3.83 (3.74-3.91) | 3.77 (3.68-3.84) | 3.44 (3.36-3.52) | 3.18 (3.11-3.26) | 0.96 (0.96-0.97) |
| Antiplatelets | 41.6 (41.4-41.8) | 38.7 (38.5-38.9) | 35.9 (35.7-36.0) | 22.0 (21.8-22.2) | 18.2 (18.1-18.4) | 16.6 (16.5-16.8) | 15.3 (15.2-15.4) | 0.82 (0.82-0.82) |
| Antiplatelets (no aspirin) | 11.8 (11.6-11.9) | 11.1 (11.0-11.2) | 10.5 (10.4-10.6) | 10.9 (10.8-11.0) | 10.4 (10.2-10.5) | 9.74 (9.63-9.85) | 9.14 (9.03-9.26) | 0.96 (0.96-0.97) |
| Aspirin | 29.8 (29.6-30.0) | 27.7 (27.5-27.9) | 25.5 (25.2-25.7) | 11.3 (11.1-11.4) | 8.08 (7.96-8.19) | 7.05 (6.94-7.16) | 6.29 (6.19-6.49) | 0.75 (0.74-0.75) |

DOAC: direct oral anticoagulant; OAC: oral anticoagulant; PE: pulmonary embolism.
